# Neurocognitive and Psychosocial Outcomes in Pediatric Brain Tumor Survivors

**DOI:** 10.3390/bioengineering5030073

**Published:** 2018-09-11

**Authors:** Peter L. Stavinoha, Martha A. Askins, Stephanie K. Powell, Natasha Pillay Smiley, Rhonda S. Robert

**Affiliations:** 1 The University of Texas MD Anderson Cancer Center, Houston, TX 77030, USA; maskins@mdanderson.org (M.A.A.); rrobert@mdanderson.org (R.S.R.); 2 Ann and Robert H. Lurie Children’s Hospital of Chicago and Northwestern Feinberg School of Medicine, Chicago, IL 60611, USA; SkPowell@luriechildrens.org (S.K.P.); NPillaySmiley@luriechildrens.org (N.P.S.)

**Keywords:** pediatric brain tumor, late effects, neurocognitive, cognitive, psychosocial, survivorship

## Abstract

The late neurocognitive and psychosocial effects of treatment for pediatric brain tumor (PBT) represent important areas of clinical focus and ongoing research. Neurocognitive sequelae and associated problems with learning and socioemotional development negatively impact PBT survivors’ overall health-related quality of life, educational attainment and employment rates. Multiple factors including tumor features and associated complications, treatment methods, individual protective and vulnerability factors and accessibility of environmental supports contribute to the neurocognitive and psychosocial outcomes in PBT survivors. Declines in overall measured intelligence are common and may persist years after treatment. Core deficits in attention, processing speed and working memory are postulated to underlie problems with overall intellectual development, academic achievement and career attainment. Additionally, psychological problems after PBT can include depression, anxiety and psychosocial adjustment issues. Several intervention paradigms are briefly described, though to date research on innovative, specific and effective interventions for neurocognitive late effects is still in its early stages. This article reviews the existing research for understanding PBT late effects and highlights the need for innovative research to enhance neurocognitive and psychosocial outcomes in PBT survivors.

## 1. Introduction

Approximately 2970 children and 1170 adolescents are diagnosed with brain and central nervous system tumors in the United States annually [1]. As survival rates following pediatric brain tumor (PBT) increase with improvements in detection and intervention, focus has increased on monitoring and managing the late effects of both disease and treatment (i.e., delayed emergence of neurocognitive, emotional and socioemotional sequelae). It is estimated that as many as 40 to 100% of survivors demonstrate impairment in at least one neurocognitive domain [2] and adult survivors of PBT report the poorest health-related quality of life among all childhood cancers [3]. Neurocognitive and psychosocial late effects are associated with lower high school and college graduation rates and increased likelihood of unemployment [4,5,6,7,8,9], all of which may adversely impact quality of life.

Late effects of treatment for PBT typically emerge in the first few years following treatment and clinically may range from mild performance difficulties that are easily accommodated to severe deficits in functioning that result in the ongoing need for support into adulthood. Here we provide a broad overview of neurocognitive and psychosocial late effects of treatment for PBT, including discussion of significant risk factors and pathophysiology of late effects, a summary of intervention paradigms and discussion of future opportunities to improve outcomes for survivors.

## 2. Factors Related to Expression of Late Effects

To understand the mechanisms of late effects, a host of factors must be considered. It is important to recognize that isolating the influence of any one variable among the multiple confounding and interacting variables is a consistent challenge in late effects research. With that caveat, factors with documented relationships to outcomes include tumor variables, treatment paradigms and potential moderating variables related to individual patient characteristics and environmental factors.

### 2.1. Tumor Variables

Tumor size has been associated with lower overall intelligence [1]. Higher risk pathology, such as medulloblastoma, has been associated with poorer neurocognitive outcomes, evident on measures of intelligence, aspects of attention, working memory and processing speed [2,3]. Tumor location is integral, in part due to associated complications. For example, 70–80% of children with posterior fossa tumors develop obstructive hydrocephalus, with approximately 30% requiring cerebrospinal fluid diversion via ventriculoperitoneal shunt or endoscopic third ventriculostomy [4]. Hydrocephalus has been shown to independently correlate with neurocognitive deficits even with otherwise uniform chemotherapy and radiation treatment [5] and is associated with poorer long term intellectual outcomes, regardless of tumor type [6]. Some evidence suggests that children with infratentorial tumors have greater neurocognitive burden than those with supratentorial tumors [7].

### 2.2. Treatment Variables

Advances in neurosurgical techniques over the last few decades have led to improved histologic diagnosis and decreased morbidity and mortality. Some tumors require only neurosurgical intervention. Still, studies suggest at least some short-term risk for neurocognitive deficits within the first year post surgery [8,9,10]. For example, even with refined neurosurgical practice, the post-surgical complication of posterior fossa syndrome (also known as cerebellar mutism) still occurs in up to 31% of children with infratentorial tumors [11]. This poorly understood entity has been attributed to disruption of cerebello-thalamo-cerebral pathways and is characterized by a unique constellation of symptoms that emerge approximately 24–48 h after surgery, including diminished speech, ataxia, emotional/behavioral lability and apathy. Although the speech and neurologic sequelae often improve with time and rehabilitation, recent evidence suggests worse overall neurocognitive outcomes for PBT survivors who experienced posterior fossa syndrome relative to those who did not [12].

Cranial radiation therapy (CRT) is often considered the most significant treatment-related risk factor for development of neurocognitive late effects [13]. CRT has been associated with significant declines in multiple neurocognitive domains that may continue for years post treatment [14]. Changes to white matter have received much attention as a mechanism of neurocognitive decline following radiation therapy, including decreased normal appearing white matter [15,16,17]. Cranial radiation also affects the growth of new neurons in the hippocampus [18] and decreased hippocampal volume has been associated with specific memory deficits [19] in PBT survivors. Further, working memory performance has been specifically associated with white matter integrity within cerebello-thalamo-cerebral pathways [20].

Effects of chemotherapy alone are difficult to isolate in the context of other treatment paradigms such as surgery and CRT, as well as in the presence of other tumor related variables and complications as reviewed above. While chemotherapy is thought to be less toxic than radiation therapy, specific chemotherapy agents are known to carry direct risk for cognitive impairment [21,22,23] as well as an indirect risk related to ototoxicity [24,25]. Further, concomitant chemotherapy and radiation appears to result in greater cognitive and educational burden compared to CRT alone [26,27].

### 2.3. Individual Patient and Environmental Characteristics

Age at diagnosis and treatment, as well as time since treatment, moderate neurocognitive outcomes in PBT survivors. Specifically, younger age at diagnosis and treatment has been associated with lower intellectual ability, processing speed, working memory, aspects of attention and academic performance [2,3,28]. In fact, a recent meta-analysis found time since treatment more predictive of overall intelligence than treatment modality in PBT survivors [13]. Further, higher levels of cognitive ability prior to treatment have been associated with greater declines in functioning after PBT treatment [2,28].

Additionally, environmental factors including low socioeconomic status and high stress levels may increase risk for poor neurocognitive and psychosocial outcomes [29,30,31,32,33]. It is well established that survivors of childhood cancer miss a considerable amount of school even after treatment is complete [34], though there is a paucity of research investigating the impact of this and other patient-specific experiential factors on neurocognitive outcome. There have been mixed research findings regarding the impact of gender on cognitive outcomes. While some studies have suggested female medulloblastoma survivors are at higher risk for poorer neurocognitive outcomes than males [28,35,36], others have failed to replicate this finding [5,37,38].

**Figure 1:**
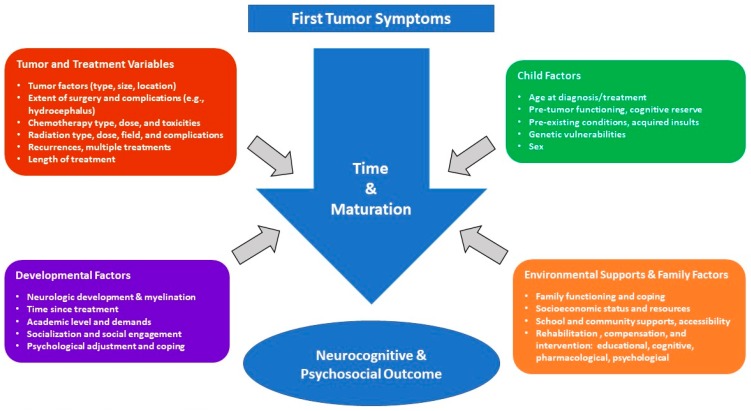
Factors affecting outcome in PBT survivors.

Multiple variables contribute to—and moderate—neurocognitive outcomes in PBT survivors as depicted in Figure 1 adapted from Dennis [39] and Baum, et al. [40]. Moreover, with increasing survivorship and risk-mitigating modifications to treatment regimens, it has become increasingly important to assess neurocognitive and psychological outcomes on an individual level. Several organizations have published psychosocial standards of care for long-term PBT survivors, including the Children’s Oncology Group [41] and the Psychosocial Standards of Care Project for Childhood Cancer [42]. Proposed clinical services range from clinical surveillance to comprehensive neuropsychological evaluations [40].

## 3. Late Effects of PBT

### 3.1. Neurocognitive Outcomes

Early studies of neurocognitive outcomes for survivors of PBT focused on global cognitive dysfunction, typically investigating the impact of brain tumors and their treatment on IQ scores and their trajectory over time. More recently, studies have identified specific cognitive functions at greatest risk, believed to represent core deficits that contribute to broader difficulties.

#### 3.1.1. Intellectual Functioning

Declines in IQ are evident in PBT survivors as early as the first year following diagnosis and treatment [43], with potential gradual progression over the next 5 to 7 years [44,45,46]. Few studies have followed survivors longer; however, a handful of studies exploring adult survivors have reported IQs approximately one full standard deviation below healthy controls or the population mean [47,48,49]. Overall intelligence, as well as language-based abilities and non-language abilities, have all been shown to be impacted, with greatest effect sizes for nonverbal functions [13,48,50]. This may be related to the demands commonly administered nonverbal tasks place on visual attention, spatial processing and timed performance [13].

Notably, the decline in IQ scores evident in PBT survivors is related to a failure to make age-appropriate gains over time rather than an actual loss of skill. This was demonstrated in a seminal study [51] in which survivors of medulloblastoma achieved gains in raw scores but only at 49% to 62% the rate of their healthy same-age peers.

The impact of pediatric brain tumors on IQ appears mediated by age, disease and treatment variables. Children diagnosed and treated at a young age (<7) are at greatest risk [52,53], with a potentially more rapid initial decline that plateaus compared to older children, who display a slower, more protracted course [14,52]. Multiple studies have established CRT to carry substantial risk to IQ, mediated by dose, delivery and target [44,45,54,55]. Proton beam radiation (PBRT) has been proposed to carry less neurocognitive risk relative to traditional CRT [56]. Preliminary evidence suggests potential sparing of cognitive and academic functions [57], particularly with focal PBRT [58,59].

#### 3.1.2. Core Deficits—Attention, Processing Speed, & Working Memory

Problems with attention, working memory and processing speed are some of the most commonly reported findings in studies of neurocognitive late effects [2,46,48,60,61]. In a recent meta-analysis, Robinson et al. [50] reported medium to large negative effect sizes for survivors of posterior fossa tumors in multiple cognitive domains, with the largest in attention (Hedges’ g = −1.69) and processing speed (g = −1.40). Moreover, numerous studies have demonstrated a pattern of declining processing speed and working memory scores over time [2,14,45,51,62].

Notably, slowed processing speed is very common in long-term PBT survivors, regardless of tumor type [62]. Processing speed is also suggested to be the most significantly impacted cognitive domain subsequent to treatment for medulloblastoma [2]. Within developmental models, processing speed is conceptualized as a foundational capacity upon which other more complex cognitive abilities are dependent [49,63,64]. For example, analyses have demonstrated age-related gains in processing speed to account for the vast majority of age-related gains in working memory [64]; in addition, these tandem gains in processing speed and working memory occur with a corresponding improvement in intellectual ability. 

Given this, it has been proposed that treatment-related deficits in processing speed, attention and working memory are the driving force behind the slowed rate of cognitive development and academic achievement observed in PBT survivors [2,31,49,51,65,66]. Some work has been done developing and evaluating specific interventions targeting these core deficits, although this remains an area of need for continued investigation.

#### 3.1.3. Other Cognitive Functions

Meta-analyses of cognitive late effects in pediatric brain tumor survivors [48,50] have revealed large effect sizes in visually-based tasks, including nonverbal IQ and visual-spatial processing (Hedges’ g = −0.88 to −1.29), as well as medium effect sizes in visual memory (g = −0.68). Studies in other cancers suggest exposure to cranial radiation carries risk for visual-spatial deficits [63]. However, a recent study looking explicitly at children with cerebellar low-grade gliomas requiring surgery alone suggests cerebellar involvement may be sufficient to cause visual-spatial impairment [67].

Executive functions refer to cognitive processes necessary for self-regulation and self-management of thinking, emotions and behavior, ranging from basic attentional and inhibitory control to more complex cognitive flexibility, set shifting and planning. Related deficits have been shown in PBT survivors relative to typically developing peers, both in terms of performance on clinical measures [68,69] and per standardized parent report [70,71]. Interestingly, PBT survivors may have limited awareness of their deficits, characterized by poor metacognition and unrealistic expectations of their abilities [46]. Executive deficits have been specifically associated with poorer long-term outcomes including lower rates of high school graduation and full employment [47,72].

Although less studied than other cognitive domains, memory problems have been demonstrated in PBT survivors across tumor type [73,74,75,76,77,78]. The majority of children with medulloblastoma demonstrate some level of memory impairment; survivors of astrocytoma may be comparatively less impaired but still perform below normal control groups [74,75]. Memory difficulties persist at least into adolescence and young adulthood following PBT [79]. While verbal memory appears more impaired than visual memory [48,50], longitudinal progressive decline has been observed in visual but not verbal memory [14,80].

Language is another area of known risk following treatment for PBT that has not been extensively researched. Meta-analyses have reported medium to large negative effect sizes in PBT survivors, in both general language abilities (Hedges’ g = −0.93 & −0.8) and verbal reasoning (g = −0.74 & −0.68) [48,50]. Cerebellar tumors in particular present with a range of speech and language deficits, including dysfluencies, slowed speech and reduced verbal abilities [81,82], associated with posterior fossa syndrome as discussed above.

PBT survivors are also at risk for sensory and motor impairments that can have a negative downstream impact on learning, academics, communication and social success. For instance, survivors may display early or delayed onset hearing loss attributed to the ototoxic effects of specific chemotherapy agents, as well as potential radiation-related damage to auditory structures [24,25,83].

### 3.2. Psychosocial Outcomes

Even though psychological problems after PBT have received less research attention than neurocognitive dysfunction, evidence suggests survivors are at greater risk for depression, anxiety, suicidal ideation and behavior problems relative to the general population [84]. Sense of well-being [85], family functioning [86], parent-child health related communication [87] and social involvement [88] have all been implicated as areas of risk after PBT. 

Psychological problems and their prevalence rates are highly variable across samples, which impedes conclusive statements regarding patterns of psychological outcome [89]. Social deficits are well established in this population, including low social competence relative to typically developing children, siblings and survivors of non-CNS cancers [90,91]. Causation is unclear, although potential contributors include level of social skill development, functional or sensorimotor deficits, separation from peers and social networks, temperament and specific neurocognitive deficits such as decreased cognitive ability and attention [92,93].

The examination of individual differences and their impact on PBT survivors’ psychological health has received some attention. As an example, neurocognitive dysfunction has been consistently associated with emotional and behavioral health [94,95,96]. Particular aspects of neurocognitive dysfunction, including executive function problems, present exponentially greater risk for emotional and behavioral health concerns in PBT survivors [97].

A recent conceptualization of the impact of childhood cancer on neurodevelopmental trajectory posits that the experience of childhood cancer is an early threat exposure that impacts psychological functioning and neural development [98] which helps unify the importance of addressing both psychosocial and neurocognitive late effects of PBT.

## 4. Interventions to Support PBT Survivors

Academic accommodations and modifications remain the primary educational support for academic difficulties resulting from neurocognitive late effects of tumor and treatment [99]. Indeed, special education utilization rates are especially high for this population relative to other types of childhood cancer [100]. Ongoing surveillance for neurocognitive and academic difficulties is considered standard of care and helps to inform academic supports and educational planning [41,42]. Career and vocational counseling may be helpful for PBT survivors who often face difficulties obtaining and maintaining employment when impacted by neurocognitive late effects [101,102].

In addition to educational supports, a number of cognitive training paradigms have targeted aspects of cognitive performance and academic achievement by attempting to enhance commonly affected functions including attention, working memory and processing speed [103]. Among the few studies that exist, results have largely been equivocal in terms of positive impact on brain tumor survivors’ academic performance and outcome [42,103].

One PBT targeted paradigm utilized drill-oriented practice, metacognitive and learning skills acquisition and cognitive behavioral therapy [104,105] focused on improving attention and academic achievement. Statistically significant improvements were observed in a number of clinical measures. However, the relevance of these clinical test and rating improvements to the children’s everyday performance was not established and this is unfortunately a common theme in research intervention programs targeting PBT late effects [103].

Recent attention has been devoted to computer-based training with the Cogmed [106] program to improve working memory using computer exercises along with regular coaching and support. Studies have suggested the intervention is feasible and acceptable in pediatric cancer survivors [107]. Randomized trials have shown performance improvements on clinical testing [108] and such improvements may be durable for months after the intervention [109]. However, studies supporting this program have not demonstrated specificity of computer-based training as the specific agent of improvement while controlling for level of support and coaching offered to participants [110]. More compelling evidence is needed before this intervention should be broadly recommended as efficacious in the PBT survivor population [103].

In addition to efforts focused on remediating specific neurocognitive deficits, other methods have addressed improving outcomes more globally in PBT survivors. For example, researchers have focused on indirect and contextual methods rooted in the premise that improving controllable external variables may hold promise for optimizing performance of brain-based functions that otherwise may not be amenable to direct intervention. For instance, training parents in behavioral modification, cognitive instructional methods and compensatory strategies to allow for ongoing intervention in the child’s natural environment showed some efficacy in improving academic outcomes and warrants further attention [111]. As another example, a recent randomized study isolated improvements in situational motivation as associated with improved academic performance [112]. Situational and intrapersonal factors such as level of intrinsic achievement motivation and responsivity to external incentive may have a role in improving academic performance in PBT survivors. Finally, targeting health-related behaviors such as exercise, has shown promise in neural recovery and neurocognitive improvement and deserves further attention [113].

Pharmacological interventions to address neurocognitive late effects have been used with PBT survivors. Stimulant medications have been shown to improve aspects of attention in survivors but not intellectual functioning or academic skills [114]. In a small pilot study, donepezil—an acetylcholinesterase inhibitor—was shown to be feasible and to improve executive functioning and memory in childhood brain tumor survivors [115], justifying a more rigorous placebo controlled randomized trial. Modafinil has been examined as a possible medication to improve fatigue, cognitive functioning and mood in adult patients with primary brain tumors but its benefits did not exceed that of the placebo control group [116]. Pharmacologic prophylaxis to diminish neurotoxicity and preserve neurocognitive function after PBT treatment has shown preliminary utility in adults undergoing whole brain radiation [117] but no such prophylactic treatments have yet been systematically studied in children.

Finally, psychological interventions have demonstrated efficacy in ameliorating PBT survivors’ behavioral and emotional health problems [118,119]. However, psychological referral standards have yet to be established and there is a clear disconnect in that the number of reported concerns far exceeds the frequency of referral for psychological services [96,120]. 

**Figure 2:**
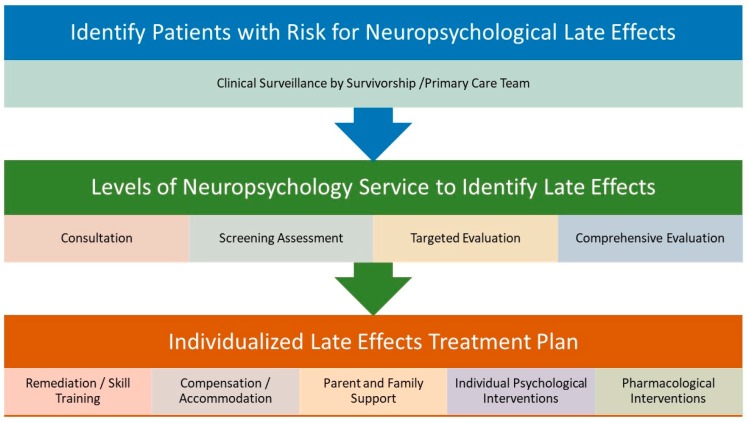
Clinical management of neuropsychological late effects after PBT.

Figure 2 provides an overview of the recommended clinical management of neuropsychological late effects of PBT survivors, inclusive of ongoing clinical surveillance through individualized treatment planning. Because of the heterogeneity of factors affecting outcomes and the diversity of outcomes themselves, there currently is not a singular consensus pathway, timeline, or group of identified supports recommended for all patients. Ongoing surveillance for neurocognitive late effects is essential to engaging subsequent clinical neuropsychological assessment and treatment planning to optimize outcomes for PBT survivors.

## 5. Conclusions

PBT outcomes research is challenging for numerous reasons and it is within this context that the bulk of neurocognitive and psychosocial outcomes research should be understood. Small base rates of specific pediatric tumor types have often resulted in small research samples. A common amelioration of this challenge has been to include mixed types of pediatric cancers and treatment paradigms, though this then contributes to variable rates of reporting of things like psychological difficulties specific to tumor variables and treatment patterns [121]. Accruing patients over long periods of time is another potential remedy, though changes in treatment-related variables (e.g., changes in chemotherapy and/or radiation protocols) may result in incomparable samples over time. Non-medical and demographic factors known to correlate with cognitive and psychosocial functioning (e.g., family functioning, socioeconomic factors) are often unaccounted for and the unique nature of PBT research complicates identification of an appropriate “control” group. While the majority of PBT outcomes studies are cross-sectional, those that are longitudinal often suffer from a lack of clearly defined or valid baseline to which later results can be compared [122]. Finally, the needs of patient care are sometimes at odds with scientific rigor, as providing clinically useful information to families and providers may not align with consistency of data gathering.

While many challenges exist in studying this population, there have been improvements and innovations over the past several decades in terms of treatment paradigms to spare neurocognitive and psychosocial functioning in PBT survivors that have led to meaningful and even dramatic improvements in long-term outcome. Largely these improvements have resulted from refined treatment protocols that have reduced neurotoxicity of treatments for PBT and unfortunately less progress has been achieved in terms of intervention to improve late effects experienced by PBT survivors.

Nonetheless, as summarized above, clinicians and researchers alike should note the development of several promising domains that warrant more attention and provide potentially fruitful topics for future clinical research. While educational support through schools is considered standard of care, there is significant opportunity to improve educational programming and support to optimize academic outcomes. Further, novel intervention paradigms have shown some promise in recent years including direct training of neurocognitive functions affected by treatment for PBT. Individual factors such as intrinsic motivation and resilience are now being considered in terms of their relationship to neuropsychological late effects. Recent work has demonstrated potential efficacy of parent and family support as a way to ameliorate late effects. Pharmacological interventions have only recently been explored and clearly there are opportunities for collaborative clinical research to investigate efficacy of medications to improve neurocognitive function after treatment for PBT. Efforts investigating the impact of health-related behaviors such as nutrition and exercise on outcomes from PBT and its treatment are in their infancy and additional research in this area may help identify cost effective and readily accessible ways to improve neurocognitive functions in the PBT population. Finally, the role of preventative methods to reduce late effects burden is only now being explored and may represent a significant opportunity to improve outcomes for PBT survivors.

Optimal clinical management of neuropsychological late effects after treatment for PBT begins with awareness of the need to monitor this population at high risk for neuropsychological deficits. Unfortunately access to appropriate neuropsychological surveillance, evaluation and intervention remains inconsistent for PBT survivors [41,42]. Moreover, we are still in the beginning stages of determining effective strategies for implementing proposed standards of neuropsychological care, meeting patient need within the current healthcare climate and resource constraints common in various clinical settings. Advocacy for improved access to surveillance and care for neuropsychological late effects is the shared responsibility of researchers and clinicians working with this population to bring to the fore the needs of this vulnerable population as well as to establish efficacy of new and innovative interventions to improve outcomes.

Finally, in addition to efforts to improve surveillance and intervention paradigms and access to care, innovations in the basic conceptualization of mechanisms of cognitive impairment in pediatric cancer, such as examining structural connectome organization implicated in efficiency of information processing [123], may further refine our understanding and detection of late effects of PBT and its treatment. What is clear is that preventing, managing and remediating late neurocognitive and psychosocial late effects for PBT survivors is going to require innovation and problem-solving among numerous basic and applied scientific disciplines.
